# Discovering Correlations between the COVID-19 Epidemic Spread and Climate

**DOI:** 10.3390/ijerph17217958

**Published:** 2020-10-29

**Authors:** Shaofu Lin, Yu Fu, Xiaofeng Jia, Shimin Ding, Yongxing Wu, Zhou Huang

**Affiliations:** 1 Faculty of Information Technology, Beijing University of Technology, Beijing 100124, China; linshaofu@bjut.edu.cn (S.L.); 13138109993@163.com (Y.F.); 2 Beijing Institute of Smart City, Beijing University of Technology, Beijing 100124, China; 3 Beijing Big Data Center, Beijing 100142, China; jiaxf@jxj.beijing.gov.cn; 4 Green Intelligence Environmental School, Yangtze Normal University, Chongqing 408107, China; 5 Institute of Remote Sensing and Geographical Information Systems, School of Earth and Space Sciences, Peking University, Beijing 100871, China; yongxing@pku.edu.cn

**Keywords:** multiple linear regression, COVID-19, climate correlation

## Abstract

The outbreak of Corona Virus Disease 2019 (COVID-19) has affected the lives of people all over the world. It is particularly urgent and important to analyze the epidemic spreading law and support the implementation of epidemic prevention measures. It is found that there is a moderate to high correlations between the number of newly diagnosed cases per day and temperature and relative humidity in countries with more than 10,000 confirmed cases worldwide. In this paper, the correlation between temperature/relative humidity and the number of newly diagnosed cases is obvious. Governments can adjust the epidemic prevention measures according to climate change, which will more effectively control the spread of COVID-19.

## 1. Introduction

Corona Virus Disease 2019 (COVID-19) is a global pandemic and serious threat to human health which halt the economic activities [1]. The COVID-19 outbreak has become a global public health emergency [2]. Corona Virus Disease 2019 (COVID-19) is pneumonia caused by SARS-CoV-2 infection. Since December 2019, there have been many cases of pneumonia caused by this virus infection all over the world. The COVID-19 pandemic forced many countries to implement full or partial lockdown, causing a substantial reduction in the anthropogenic activities due to being prohibited from outdoor invasion which resulted in less transportation and shutting down of industries [3]. On 11 March 2020, the World Health Organization (WHO) declared COVID-19 a world pandemic [4]. To date, COVID-19 infection has been reported worldwide and may be further transmitted by air travel [5]. Currently, many scholars are conducting research on the spatial spread of COVID-19. For example, Jelodar used LSTM Recurrent Neural Network (RNN) to carry out deep emotional classification of COVID-19 [6]; Alsaeedy used the existing cellular wireless network function to detect COVID-19 risk areas [7].

According to the data on “Baidu COVID-19 Epidemic Real-time Big Data Report”, from 15 February to 22 June, the total number of confirmed cases of the COVID-19 in the United States exceeded 2.31 million, and the cumulative number of deaths exceeded 120,000, reaching 123,053. The cumulative number of confirmed cases of COVID-19 in Brazil exceeded 1.1 million, and the cumulative number of deaths exceeded 50,000, reaching 51,271. As of 22 June, three countries in the southern hemisphere were among the top five in the daily list of newly diagnosed cases. On 22 June, there were 26,616 new cases in Brazil. Therefore, there is an urgent need to evaluate the correlation between the spread of COVID-19 and climate conditions, so as to better support the decision making and response measures and control the further spread of COVID-19.

## 2. Related Research Work

### 2.1. Research on Epidemic Situation and Climate

Since 2000, Severe Acute Respiratory Syndrome (SARS), bird flu and other epidemics have broken out successively. The SARS epidemic broke out in Guangdong, China in mid-November 2002, and then spread to other parts of China and the world. Under the action of a series of countermeasures, SARS disappeared in mid-June 2003. SARS lasted for about 7 months, and reached its peak from April to May 2003. Bird flu epidemics mostly occur in winter and spring, and they all end in late spring to early summer. SARS was first discovered in Guangdong and Hong Kong, China in 2003 with the warm climate, where the average temperature from January to February is above 10 °C. The bird flu was first discovered in Guangxi, Hubei and other provinces in 2004 with a suitable climate, while the average temperature from January to February is around 10 °C. The COVID-19 epidemic was first reported in Wuhan, Hubei Province, while the average temperature from January to February was also around 10 °C [8].

In China, the epidemics such as SARS and bird flu have the common characteristics of beginning in winter, ending in summer, and originating in southern China. These viruses have high activity and transmission capacity in low-temperature and high-humidity environment [9]. Some reference believe that the increase temperature will the virus lose its infective activity.

At present, there have been some studies on the correlation between the COVID-19 epidemic spread and climate. Zhu et al. [10] confirmed the highly significant correlation between absolute humidity and daily new COVID-19 cases using Multiple Linear Regression Model, after collecting the daily number of new cases and corresponding climate data from eight regions in four countries in South America. David et al. [11] utilized the generalized additive model (GAM) to explore the linear and non-linear relationship between the annual average temperature compensation and the confirmed COVID-19 cases in the capital city of Brazil. It was found that the daily cumulative number of confirmed cases decreased by 4.8951% when the temperature increased by 1 °C. Goswami [12] used Sen’s Slope and Man-Kendall test and generalized additive regression model (GAM) to detect the impact of daily temperature and relative humidity on incidence rate in India countries. Lowen [13], Barreca [14] and Żuk [15] pointed out that environmental temperature plays an important role in the survival and transmission of viruses.

A large number of studies reveal that temperature and humidity can affect the spread of epidemics, thus prompting this study to further explore the global impact of environmental factors on COVID-19.

At present, all the current literature selected samples are limited to local areas, which may lead to the problem that the conclusions are not universal. Therefore, this study collected the epidemic situation and meteorological data in the high incidence area of global epidemic situation, analyzed the development trend of the epidemic situation on a global scale, including more climatic conditions that may affect the spread of the virus, so as to study the climatic factors affecting the activity of SARS-CoV-2.

### 2.2. Multiple Regression Analysis

Multiple Linear Regression Models are suitable for scenarios where multiple variables affect single outcome. It can accurately measure the correlation degree and regression fitting degree of each variable, and improve the prediction model effect. In this study, climate factors have an impact on the spread of the epidemic in many aspects. As it was necessary to evaluate the correlation between various climatic factors and the spread of SARA-CoV-2. Multiple Linear Regression Models was selected for analysis.

Multiple Regression Analysis Models have been widely used in various scenarios of COVID-19. Rath [16] used Multiple Linear Regression Model to predict that the number of daily active cases in India would reach 52,290 by 15 August. Ayyoubzadeh et al. [17] used Multiple Linear Regression Method to predict the spread of COVID-19 in Iran, and found that in addition to the incidence of the previous day, factors that can effectively improve the accuracy of the prediction also include hand sanitizer usage and hand washing frequency. Kass et al. [18] analyzed the relationship between Body Mass Index (BMI) and age in patients diagnosed with COVID-19 through Multiple Linear Regression Model, and concluded that obesity may increase the infection rate of COVID-19. Yang et al. [19] estimated the early mortality of COVID-19 by linear regression model, and concluded that the mortality of COVID-19 was lower than that of coronavirus epidemic caused by SARS-CoV and MERS-CoV. Xiong et al. [20] analyzed the correlation between initial CT features and turbidity progression in COVID-19 patients by linear regression models and Spearman correlation coefficient.

## 3. Data Sources and Research Methods

### 3.1. Data Sources

The open data sets of confirmed cases published by the Center for System Science and Engineering (CSSE) of Johns Hopkins University were used in this study. The data from all over the world were collated from 22 January, the early stage of the epidemic. Considering that countries with less confirmed epidemic cases might have problems with less obvious climate characteristics, and it is necessary to avoid the problem that too few objects lead to the research results not being universal, 65 countries with more than 10,000 confirmed cases from 22 March to 22 June were selected. The total number of confirmed cases per day is subtracted from that of the previous day to get the new daily number of confirmed cases in each country, so as to reflect the epidemic transmission capacity.

The climate data in this study comes from the daily records of weather stations around the world collected by China Meteorological Data Network (http://data.cma.cn/). We selected high average monthly temperature, low average monthly temperature, sea level pressure, altitude, wind speed, rainfall, dew point temperature and relative humidity as the climatic indicators of each region from 22 March to 22 June during the epidemic periods in each region to reflect the regional weather changes.

In this experiment, 65 countries with more than 10,000 confirmed COVID-19 cases at 24:00 on 22 June were selected as experimental subjects. The number of newly diagnosed cases per day and 8 climate factors were collected for experimental analysis. Set the number of new daily confirmed cases (New) as the dependent variable y, the monthly average maximum temperature during the epidemic periods (Tmax), the monthly average minimum temperature during the epidemic periods (Tmin), sea level pressure (Sea_Pressure), Wind_Speed, Elevation, Rainfall, Dew point temperature (DP), and Relative humidity (Humidity) are respectively Arguments x1, x2, x3, x4, x5, x6, x7, x8. The samples of observation data are shown in Table 1.

### 3.2. Methodology

We chose the Multiple Linear Regression Analysis Method to analyze the correlation between the number of daily increased confirmed cases in each region and the climate indicators of the region. Firstly, the relevant Multiple Linear Regression Method was used to perform a series of verifications and establish a multiple regression equation. Then the Pearson correlation coefficient was used to evaluate the relative importance of the influence of each independent variable on the dependent variable, i.e., the correlation coefficient between each independent variable and the dependent variable. The linear relationship between them was discovered and the correlation between the observed variables was determined. The advantage of using this method is that the relationship between the variables can be clearly defined and expressed quantitatively, and the influence of different climatic factors on the number of new diagnosed cases per day can be clearly shown.

According to the selected observation variable data, the Multiple Linear Regression Models as shown in Equation (1) can be constructed.

(1) $$\begin{array}{c} y=\beta_{0}+\beta_{1}x_{1}+\beta_{2}x_{2}+\beta_{3}x_{3}+\beta_{4}x_{4}\\ +\beta_{5}x_{5}+\beta_{6}x_{6}+\beta_{7}x_{7}+\beta_{8}x_{8}+\varepsilon \end{array}$$

where *y* is the dependent variable, which represents the number of newly increased confirmed cases every day in this experiment; $x_{1}$, $x_{2}$, $x_{3}$, $x_{4}$, $x_{5}$, $x_{6}$, $x_{7}$, $x_{8}$ are independent variables, respectively representing the monthly average maximum temperature, monthly average minimum temperature, sea level pressure, wind speed, elevation, rainfall, dew point temperature and relative humidity in this experiment. $\beta_{0}$, $\beta_{1}$, $\beta_{2}$, $\beta_{3}$, $\beta_{4}$, $\beta_{5}$, $\beta_{6}$, $\beta_{7}$, $\beta_{8}$ is the unknown parameters of the corresponding independent variables; ε is called the error term, which is an unobservable random variable with a mean value of zero and a variance of $\sigma^{2}>0$, and $\varepsilon \in N(0,\sigma^{2})$. The above Linear Regression Model can be used to predict the number of new daily confirmed cases and determine the correlation between each independent variable and the dependent variable. Therefore, for different dates, *n* groups of different data can be obtained, as shown in Equation (2).

(2) $$\left\{\begin{array}{c} y_{1}=\beta_{0}+\beta_{1}x_{11}+...+\beta_{8}x_{18}+\varepsilon_{1}\\ y_{2}=\beta_{0}+\beta_{1}x_{21}+...+\beta_{8}x_{28}+\varepsilon_{2}\\ \vdots \\ y_{n}=\beta_{0}+\beta_{1}x_{n1}+...+\beta_{8}x_{n8}+\varepsilon_{n} \end{array}\right.$$

where $\varepsilon_{1},\varepsilon_{2},\cdots ,\varepsilon_{n}$ is independent of each other and $\varepsilon \in N(0,\sigma^{2})$.

The Pearson correlation coefficient is calculated on the basis of the above data. For a single independent variable, the calculation method is shown in Equation (3).

(3) $$R=\frac{\sum_{i=1}^{n}\left(x_{i}-\bar{x}\right)\left(y_{i}-\bar{y}\right)}{\sqrt{\sum_{i=1}^{n}{\left(x_{i}-\bar{x}\right)}^{2}\sum_{i=1}^{n}{\left(y_{i}-\bar{y}\right)}^{2}}}$$

R (Pearson correlation coefficient) is used to describe the correlation between two groups of different data. When the development trend of two different groups of data presents weak correlation, $0\leq \left|R\right|<0.3$. When the development trend of two different groups of data show medium correlation, $0.3\leq \left|R\right|<0.6$. And When the development trendof two different groups of data presents high correlation, $0.6\leq \left|R\right|<1.0$.

Pearson correlation coefficient was utilized to analyze the correlation between variables, and the correlation coefficient values are shown in Table 2.

In Table 2, *R* is the correlation coefficient, $R_{01}$ is the correlation coefficient between the monthly average maximum temperature (Tmax) and the number of the daily increased confirmed cases (New), and $R_{10}$ is the correlation coefficient between the daily increased confirmed cases (New) and the monthly average maximum temperature (Tmax). Because the correlation between the monthly average maximum temperature (Tmax) and the number of the daily increased confirmed cases (New) is equivalent to the correlation between the number of the daily increased confirmed cases (New) and the monthly average maximum temperature (Tmax). Therefore, in the above table, $R_{01}$ = $R_{10}$, $R_{12}$ = $R_{21}$, and so on. The closer the absolute value of the correlation coefficient to 1, the stronger the correlation between the two observed variables in the region.

### 3.3. Model Testing

After the establishment of Multiple Linear Regression Model, it is necessary to test the Linear Regression Model. In this experiment, the modified determination coefficient ${\bar{R}}^{2}$ is selected to determine the performance of the model.The formula for selecting the modified determination coefficient is shown in Equation (4).

(4) $${\bar{R}}^{2}=1-\frac{SSE/(n-k-1)}{SST/(n-1)}$$

where *SSE* represents the Sum of Squares of Residuals, and *SST* represents the Sum of Squares of Deviations, while *n* − *k* − 1 represents the degree of freedom of the Sum of Squares of Residuals, and *n* − 1 is the degree of freedom of the Sum of the Squares of Deviation. By dividing the SSE by their degrees of freedom and dividing the SST by their degrees of freedom, the influence of the number of variables on the coefficient of determination can be suppressed, and the fitting degree of the linear regression model for the relationship between variables can be better reflected. The closer the correction coefficient is to 1, the higher the fitting degree of the relationship between variables is, and the more accurate the model effect is.

(5) $$R^{2}=\frac{SSR}{SST}=\frac{\sum_{i=1}^{n}{\left({\hat{y}}_{i}-\bar{y}\right)}^{2}}{\sum_{i=1}^{n}{\left(y_{i}-\bar{y}\right)}^{2}}$$

The difference between the modified determination coefficient ${\bar{R}}^{2}$ and the coefficient of determination $R^{2}$ is that a penalty term is introduced. The function of the penalty term is that our coefficient of determination can be increased only when variables that are really helpful for analysis are introduced, effectively solving the problem of As the number of independent variables in the model increases, the coefficient of determination also gradually increases.

In order to ensure that there are enough data for model training, 70% of the observation data is selected as the training set, and the remaining 30% of the observation data are used as the test set. The climate data of the day in the test set is input into the establish multiple linear regression model, and the predicted value of the number of new confirmed cases on that day is obtained, and compare with the actual observation value of the day. This is to test the constructed Multiple Linear Regression Model is in line with the actual situation.

## 4. Experimental Results and Discussion

### 4.1. Regression Analysis between the Number of New Daily Confirmed Cases and Climate Variables

After data processing with Multiple Linear Regression Models, the fitting function between the number of new daily confirmed cases and each climate variable is obtained. Among the 65 linear regression models (each model for a country), the correlation coefficient of 42 models were greater than 0.5, showing that these models have good fitting effect. Countries with the top six number of confirmed cases as of 22 June were selected for display. Table 3 and Table 4 shows the Multiple Linear Regression Model constructed by the training set of data from United States, Brazil, India, Mexico, South Africa and Peru.

**Figure 1:**
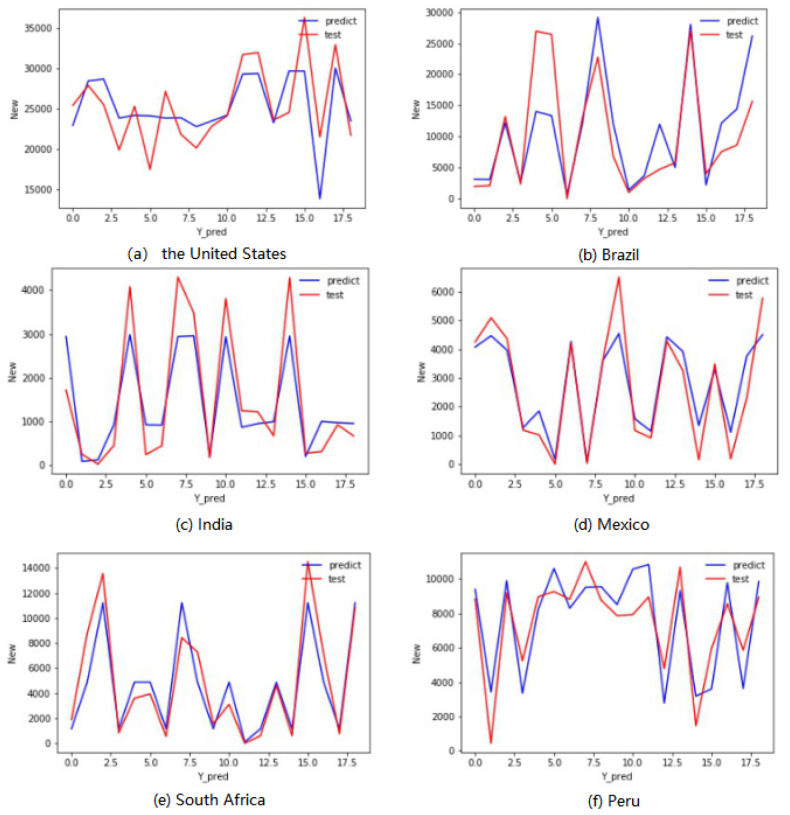
Comparison of test sets and model predictions in Different Countries.

**Figure 2:**
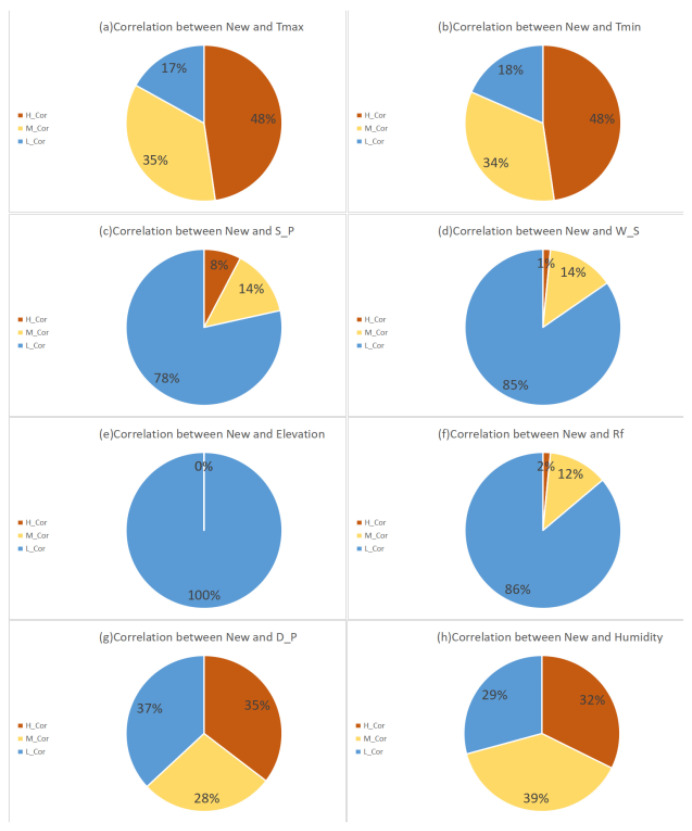
Correlations between the number of daily new confirmed cases and climate variables.

**Figure 3:**
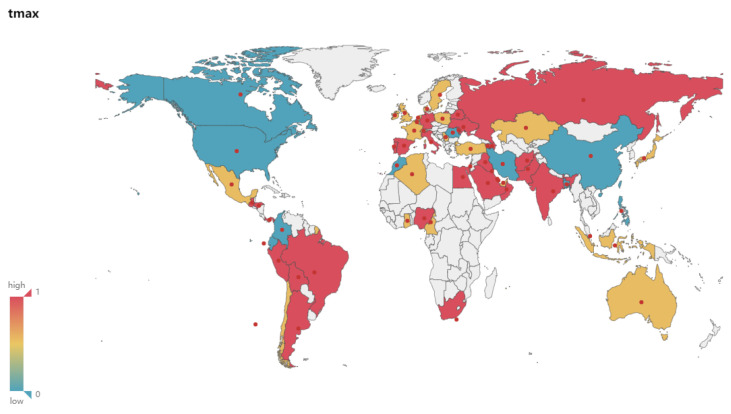
Correlation between New and Tmax.

Eight climatic parameters were inputted into the established Multiple Linear Regression Model to calculate the daily number of new confirmed cases. The results were compare with the daily number of new confirmed cases in the observation, to detect whether the Multiple Linear Regression Models can effectively reflect the relationship between the daily number of new confirmed cases and the climate data. The prediction of daily number of new confirmed cases provides a theoretical basis. Figure 1, Figure 2 and Figure 3 show the comparison between test set and model prediction of the United States, Brazil, India, Mexico, South Africa and Peru; The red curve is the observation data of the test set, and the blue curve is the daily increased number of confirmed cases predicted by the constructed multiple regression model.

According to Figure 1, it can be noted that the Multiple Linear Regression Model constructed by the experiment fits with the observation data, and can predict the number of new confirmed cases every day based on the relevant climate data.

### 4.2. The Correlation between the New Confirmed Case Number and Climate in Different Countries

We explore the relationship between the daily number of new confirmed cases (New) and climate data in different countries, and select the countries with the top six number of confirmed epidemic cases as of 24:00 on 22 June for demonstration. The correlation coefficient between the daily number of the confirmed cases and the climate data is shown in Table 5.

As can be seen in Table 5, the correlation coefficient between New of countries and each climatic variable illustrates significant correlations between New and Tmax, Tmin, Humidity.

1. For Tmax and Tmin, four of the top six countries in the total number of confirmed cases showed a strong correlation, including Brazil, India, Mexico and Peru. In addition, South Africa showed a moderate correlation, while the United States, which had the largest number of confirmed cases, showing a weak correlation.
2. For Humidity, four of the top six countries in the total number of confirmed cases showed strong correlation, including Brazil, India, Mexico and South Africa, while the United States showed moderate correlation, and Peru showed weak correlation.

### 4.3. The Correlation between the Number of New Daily Confirmed Cases and Various Climate Variables in Different Countries

In the 65 countries selected in this experiment, the experimental results of the correlation coefficient between the New and Various Climate Parameters are shown in Figure 2.

**Figure 4:**
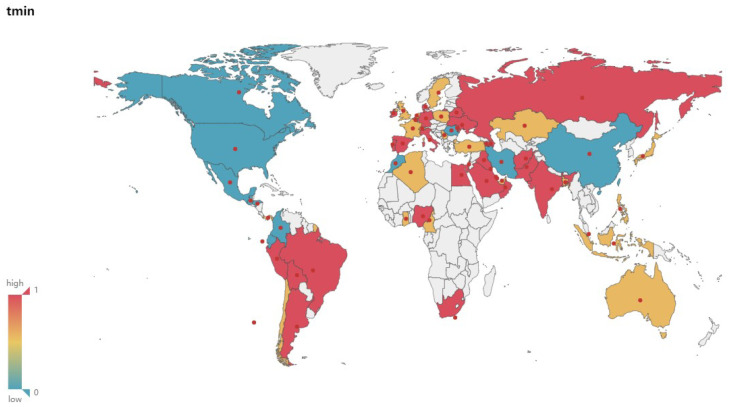
Correlation between New and Tmin.

1. The New and Tmax generally show a medium or high correlation. There are 54 countries where the New and Tmax show medium or high correlations, accounting for about 83%. Among them, there are 31, 23, and 11 countries showing high, medium, and low correlations respectively, accounting for about 48%, 35%, and 17% respectively, as shown in Figure 4a.
2. The New and Tmin generally show a medium or high correlation. There are 53 countries where the New and Tmin show medium or high correlations, accounting for about 82%. Among them, there are 31, 22, and 12 countries showing high, medium, and low correlations respectively, accounting for about 48%, 34%, and 18% respectivle, as shown in Figure 4b.
3. There is a low correlation between the New and Sea Level Pressure overall. There are only 14 countries where the New and Sea Level Pressure show medium or high correlations, accounting for about 22%. Among them, there are 5, 9, and 51 countries showing high, medium, and low correlations respectively, accounting for about 8%, 14%, and 78% respectively, as shown in Figure 4c.
4. There is a low correlation between the New and Wind Speed overall. There are only 10 countries where the New and Wind Speed show medium or high correlations, accounting for about 15%. Among them, there are 1, 9, and 55 countries showing high, medium, and low correlations respectively, accounting for about 1%, 14%, and 85% respectively, as shown in Figure 4d.
5. There is a low correlation between the New and Elevation. There is no country where the New and Elevation show medium or high correlation, while all countries show a low correlation, as shown in Figure 4e.
6. There is a low correlation between the New and Rainfall overall. There are only 9 countries where the New and Rainfall show medium or high correlations, accounting for about 14%. Among them, there are 1, 8, and 56 countries showing high, medium, and low correlations respectively, accounting for about 2%, 12%, and 86% respectively, as shown in Figure 4f.
7. The New and the Dew Point Temperature generally show a medium or high correlation. There are 41 countries where the New and the Dew Point Temperature show a medium or high correlations, accounting for about 63%. Among them, there are 23, 18, and 24 countries showing high, medium, and low correlations respectively, accounting for about 35%, 28%, and 37% respectively, as shown in Figure 4g.
8. The New and Relative Humidity generally show a medium or high correlation. There are 46 countries where the New and Relative Humidity show medium or high correlations, accounting for about 71%. Among them, there are 21, 25, and 19 countries showing high, medium, and low correlations respectively, accounting for about 32%, 39%, and 29% respectively, as shown in Figure 4h.

Based on the above analysis, the following further inferences can be drawn:

1. The activity of the COVID-19 have little correlation with Elevation, Sea Level Pressure, Wind Speed, and Rainfall. The New in all selected 65 countries shows low correlations with Elevation. Only 22% of the selected countries have medium or high correlations between the New and Sea Level Pressure, and only 8% of the countries show high correlations. Only 15% of the countries have medium or high correlations between the New and Wind Speed, and only 1% of the countries show high correlations. 14% of the countries have medium or high correlations between the New and Rainfall, and only 2% of the countries show high correlations.
2. The activity of the COVID-19 is correlated with Tmax, Tmin, Dew Point Temperature and, Relative Humidity. 82% of the selected 65 countries have medium or high correlations between the New and Tmax or Tmin, and 48% of them have high correlations. 71% of the countries have medium or high correlations between the New and the Dew Point Temperature or Relative Humidity, and about 32% of them show high correlations.
3. The activity of the COVID-19 is mainly related to Temperature and Humidity. Since both Tmax and Tmin belong to air temperature, and the Dew Point Temperature can be obtained from the Relative Humidity and Temperature [21]. It is inferred that the activity of the COVID-19 is mainly related to temperature and humidity. It is worth noting that the temperature and humidity compared with the correlation between the number of new daily confirmed cases, more countries show medium or high correlations between temperature and the number of new daily confirmed cases. The temperature seems to have a more obvious impact on virus activity. However, the correlation between the number of new daily confirmed cases and humidity should not be ignored. It is necessary to consider the impact of climate factors on the spread of the epidemic in combination with temperature and humidity.

### 4.4. Geospatial Analysis of the Correlation between the New and Climate Variables

**Figure 5:**
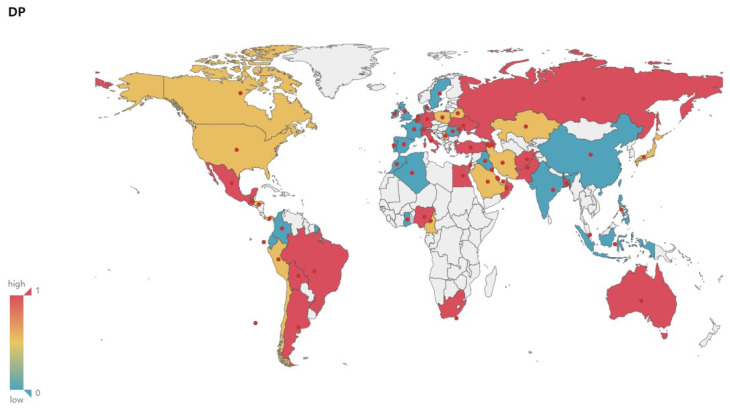
Correlation between New and DP.

**Figure 6:**
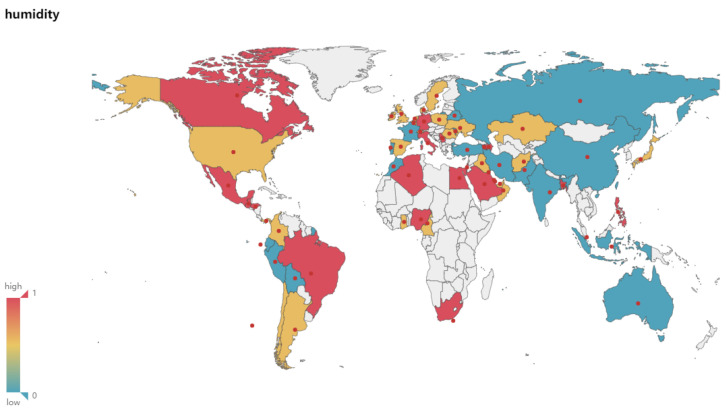
Correlation between New and Humidity.

Figure 3, Figure 4, Figure 5 and Figure 6 show the maps of the correlation between New and Tmax, Tmin, Relative Humidity, verifying and demonstrating the analysis and inference above on the whole. In the maps, red color indicates high correlation, yellow color indicates medium correlation, and blue-green color means low correlation, while white color means unselected countries. We select most countries in the world for analysis, trying to reveal the impact of various climate factors on the activity of COVID-19 in the global range, and effectively reduce the risk of erroneous conclusions because the occasional weather conditions in individual countries are similar to the climatic conditions related to virus activity, so that the experimental results are more reliable and universal. Integrating with the spatial visualization analysis in Figure 3, Figure 4, Figure 5 and Figure 6, we further analyze and infer the following points of view:

1. The intervention of epidemic prevention and control measures can restrain the influence of climatic factors on the spread of the epidemic. China, where the epidemic broke out in January, shows a low correlation between the New and various climate factors. In the analysis of Israel, South Korea, and Singapore, which had good epidemic prevention and control, the correlation analysis between the New and various climate factors also shows a low correlation. Therefore, this article speculates that government interventions in epidemic prevention and control measures can effectively reduce the spread of the epidemic and restrain the impact of climate on the spread of the epidemic.
2. At the stage of a pandemic, climate factors are not enough to restrain the spread of the epidemic. As of 22 June, the United States, which has the largest number of confirmed cases of the epidemic, does not have a high correlation between the New and Temperature and Humidity. This article speculates that the United States shows a low correlation because it was already in the pandemic stage. The main reason affecting the number of new confirmed cases daily is the large number of confirmed cases and their interpersonal and social contact. In the case of a large number of confirmed cases and failure to effectively prevent interpersonal communication, Climate factors have a low impact on the transmission speed of the epidemic. The impact of temperature and humidity on the spread of COVID-19 is not enough to completely suppress the pandemic.
3. Temperature and Humidity in tropical areas have a more obvious impact on the spread of the epidemic. Cases of COVID-19 have also been confirmed in the equatorial Africa and Amazon tropical rainforest. Countries in the relevant regions such as Brazil, Colombia, Ecuador, Nigeria and other countries have reported moderate or above correlation between the numbers of new confirmed cases and temperature, humidity. It can also be seen from the map that more countries in the equator and South America show medium or high correlation. This article speculates that temperature and humidity in the equator of Africa and the Amazon rainforest have a greater influence on the spread of the epidemic.

## 5. Discussion

According to the research results, temperature and humidity have a high correlation with the activity of SARS-CoV-2. Other literature shows that in addition to temperature and humidity, biological gender [22], obesity rate [23], age [24], comorbidities [25] can affect the transmission of COVID-19. Therefore, the government should pay special attention to some special environment when formulating measures to prevent and control the epidemic situation, which can make SARS-CoV-2 have high activity. Countries should strengthen the control of disinfection and social isolation on the environment. The development of the epidemic can be predicted according to the changes in temperature and humidity, and corresponding prevention and control measures can be taken. According to the climate change in the area where the virus activity changes, epidemic prevention, and control measures should be strengthened when the virus activity is low, so as to suppress the trend of a virus outbreak and achieve the purpose of epidemic prevention and control. At the same time, attention should be paid to the prevention and control of the environmental factors such as temperature and humidity suitable for virus survival and transmission channels. Therefore, countries must seize the influence of climatic factors to take active measures to control the first COVID-19 epidemic when the virus activity is at a low level.

Therefore, the development of the epidemic can be predicted according to the changes of temperature and humidity, and corresponding prevention and control measures can be taken. According to the climate change in the area where the virus activity changes, the epidemic prevention and control measures should be strengthened when the virus activity is low, so as to suppress the trend of virus outbreak and achieve the purpose of epidemic prevention and control. At the same time, Attention should be paid to the prevention and control of the environmental factors such as temperature and humidity suitable for virus survival and transmission channels. Therefore, countries must seize the influence of climatic factors to take active measures to control the first COVID-19 epidemic when the virus activity is at a low level. At the same time, they should not relax the prevention and control measures to prevent the second COVID-19 epidemic caused by the increase of virus activity due to the change of climate factors.

At present, the specific mechanism of the interaction between Temperature, Humidity and Virus activity is unknown. But like influenza virus, COVID-19 can be transmitted by aerosol [26]. Casanova believes that compared with medium relative humidity (50%), COVID-19 has a greater survival rate or greater protection at high relative humidity (80%) [27]. So this research speculated that low temperature and high humidity lead to the increase of suspended solids in the atmosphere, which provides the ideal conditions for virus attachment, replication and transmission. Low temperature can also dry the mucous membrane, reduce the function of cilia, and support the survival and transmission of virus and the spread of disease [28]. Therefore, it is speculated that temperature and humidity can affect the spread of COVID-19 by affecting the spread of COVID-19. On the one hand, temperature affects the human mucous membrane to reduce the human resistance to viruses. On the other hand, high humidity can increase the quality of aerosols in the air. The concentration increases the number of aerosol particles in the air [29], which leads to an increase in the speed of virus transmission. The combination of temperature and humidity reduces human resistance to viruses and enhances the speed of virus transmission, thus showing that the speed of virus transmission is accelerated, and the number of new confirmed cases increases daily.

## 6. Conclusions and Future Work

The analysis in this paper shows that the activity of the new coronavirus has the following characteristics:

1. There is a high correlation between the activity of the COVID-19 and temperature and humidity, while temperature is more correlated with the activity of the COVID-19. Wind speed, sea level pressure, altitude, and rainfall have little effect on the spread of the epidemic.
2. The intervention of epidemic prevention and control measures can restrain the influence of climatic factors on the spread of the virus. But climatic factors alone are not enough to restrain the spread of the epidemic.
3. Temperature and humidity in tropical areas have a more obvious impact on the spread of the epidemic.

Accordingly, this paper proposes the following conclusions, recommendations for global COVID-19 prevention and control.

1. It is better for each country to take appropriate or even more stringent prevention and control measures to minimize the risk of outbreak the epidemic by taking into account the development and changes of climatic factors such as temperature and humidity in the early stage of the epidemic.
2. As time goes by, the climate changes in the northern and southern hemispheres will affect the activity of the virus. It is necessary to pay special attention to the prevention and control of the virus, so as to prevent the spread of the COVID-19 epidemic in the southern hemisphere and secondary outbreak in some parts of the northern hemisphere due to the increased activity of the virus.
3. Countries with better epidemic prevention and control or countries with less serious epidemic situation shouldn’t take it lightly. It is necessary to strictly control various public areas to prevent the risk of re-outbreak caused by the enhancement of virus activity due to the climatic factors such as temperature and humidity.

This paper assesses the correlation analysis between the spread of COVID-19 epidemic and climatic factors. The number of confirmed cases is inevitably underestimated due to different detection coverage rates of COVID-19 in different countries, and the impact of changes in policies and local prevention and control strategies on the spread of the epidemic was not assessed in this study. Therefore, in the future work these issues need to be more detailed exploration.

## Figures and Tables

**Table 1:** Observation Variable Table.

Data	New	Tmax	Tmin	Sea_Pressure	Wind_Speed	Elevation	Rainfall	DP	Humidity
03/22	$Y_{1}$	$X_{11}$	$X_{12}$	$X_{13}$	$X_{14}$	$X_{15}$	$X_{16}$	$X_{17}$	$X_{18}$
03/23	$Y_{2}$	$X_{21}$	$X_{22}$	$X_{23}$	$X_{24}$	$X_{25}$	$X_{26}$	$X_{27}$	$X_{28}$
...	...	...	...	...	...	...	...	...	...
06/22	$Y_{n}$	$X_{n1}$	$X_{n2}$	$X_{n3}$	$X_{n4}$	$X_{n5}$	$X_{n6}$	$X_{n7}$	$X_{n8}$

**Table 2:** Correlation Coefficient Table.

	New	Tmax	Tmin	Sea_Pressure	Wind_Speed	Elevation	Rainfull	DP	Humidity
New	1.00	$R_{01}$	$R_{02}$	$R_{03}$	$R_{04}$	$R_{05}$	$R_{06}$	$R_{07}$	$R_{08}$
Tmax	$R_{10}$	1.00	$R_{12}$	$R_{13}$	$R_{14}$	$R_{15}$	$R_{16}$	$R_{17}$	$R_{18}$
Tmin	$R_{20}$	$R_{21}$	1.00	$R_{23}$	$R_{24}$	$R_{25}$	$R_{26}$	$R_{27}$	$R_{28}$
Sea_Pressure	$R_{30}$	$R_{31}$	$R_{32}$	1.00	$R_{34}$	$R_{35}$	$R_{36}$	$R_{37}$	$R_{38}$
Wind_Speed	$R_{40}$	$R_{41}$	$R_{42}$	$R_{43}$	1.00	$R_{45}$	$R_{46}$	$R_{47}$	$R_{48}$
Elevation	$R_{50}$	$R_{51}$	$R_{52}$	$R_{53}$	$R_{54}$	1.00	$R_{56}$	$R_{57}$	$R_{58}$
Rainfull	$R_{60}$	$R_{61}$	$R_{62}$	$R_{63}$	$R_{64}$	$R_{65}$	1.00	$R_{67}$	$R_{68}$
DP	$R_{70}$	$R_{71}$	$R_{72}$	$R_{73}$	$R_{74}$	$R_{75}$	$R_{76}$	1.00	$R_{78}$
Humidity	$R_{80}$	$R_{81}$	$R_{82}$	$R_{83}$	$R_{84}$	$R_{85}$	$R_{86}$	$R_{87}$	1.00

**Table 3:** Multiple Linear Regression Models of Some Countries.

Country	$\beta_{1}$	$\beta_{2}$	$\beta_{3}$	$\beta_{4}$	$\beta_{5}$	$\beta_{6}$	$\beta_{7}$	$\beta_{8}$	$\beta_{0}$
Brazil	−863	5554	424	826	0	0	−1409	−755	$-2.7\times e^{5}$
India	−1286	1259	−1191	0	0	$-2.1\times e^{5}$	0	$-7.6\times e^{7}$	$2.1\times e^{9}$
Peru	$4.4\times e^{14}$	$-2.3\times e^{14}$	$7.7\times e^{10}$	388	$-4.9\times e^{10}$	−6.3	$-6.9\times e^{14}$	$2.2\times e^{14}$	$-2.7\times e^{16}$
Mexico	5	368	1	0	0	−1789	−352	793	−4548
South Africa	139	−722	0	−44	0	59	188	−164	5518
US	$-3.8\times e^{9}$	$9.0\times e^{9}$	−71	84	$6.1\times e^{11}$	593	$-7.0\times e^{8}$	$8.9\times e^{8}$	$-1.4\times e^{14}$

**Table 4:** Modified Determination Coefficients of Some Countries.

Item	Brazil	India	Peru	Mexico	South Africa	US
${\bar{R}}^{2}$	0.60	0.85	0.85	0.75	0.80	0.43

**Table 5:** Correlation Coefficient between New and Climate Variables in the Top Six Countries.

Country	Tmax	Tmin	Sea_Pressure	Wind_Speed	Elevation	Rainfull	DP	Humidity
Brazil	−0.81	−0.84	0.57	0.48	$-2.0\times e^{-16}$	0.52	−0.84	−0.85
India	0.71	0.76	0.00	0.00	$-2.0\times e^{-16}$	−0.84	0.00	0.00
Peru	−0.69	−0.72	0.00	0.39	$-9.3\times e^{-17}$	−0.13	−0.38	−0.21
Mexico	0.75	0.73	−0.55	−0.31	$6.7\times e^{-16}$	−0.08	−0.84	−0.81
South Africa	−0.41	−0.37	0.13	7	$-0.1\times e^{-16}$	−0.17	−0.53	−0.80
United States	0.11	0.11	−0.02	0.19	$4.9\times e^{-16}$	0.02	−0.32	−0.46

* Due to the lack of India’s dew point temperature and relative humidity data, the correlation is set to 0 to avoid affecting the experimental results.
